# RTN3 Regulates the Expression Level of Chemokine Receptor CXCR4 and is Required for Migration of Primordial Germ Cells

**DOI:** 10.3390/ijms17040382

**Published:** 2016-04-08

**Authors:** Haitao Li, Rong Liang, Yanan Lu, Mengxia Wang, Zandong Li

**Affiliations:** State Key Laboratory for Agrobiotechnology, College of Biological Sciences, China Agricultural University, No. 2 Yuan Ming Yuan West Road, Beijing 100193, China; lihaitao_cau@163.com (H.L.); lr_@cau.edu.cn (R.L.); nan0914@126.com (Y.L.); wmx_arctic@cau.edu.cn (M.W.)

**Keywords:** primordial germ cells, RTN3, CXCR4

## Abstract

CXCR4 is a crucial chemokine receptor that plays key roles in primordial germ cell (PGC) homing. To further characterize the CXCR4-mediated migration of PGCs, we screened CXCR4-interacting proteins using yeast two-hybrid screening. We identified reticulon3 (RTN3), a member of the reticulon family, and considered an apoptotic signal transducer, as able to interact directly with CXCR4. Furthermore, we discovered that the mRNA and protein expression levels of CXCR4 could be regulated by RTN3. We also found that RTN3 altered CXCR4 translocation and localization. Moreover, increasing the signaling of either CXCR4b or RTN3 produced similar PGC mislocalization phenotypes in zebrafish. These results suggested that RTN3 modulates PGC migration through interaction with, and regulation of, CXCR4.

## 1. Introduction

Directional cell migration is crucial for embryonic development, as well as during early development and adult life. Identification of the molecular cues regulating cell migration is important for understanding the mechanism of cell movement and for developing therapies to treat diseases resulting from aberrant cell movement. An excellent model system for studying directional cell migration is that of primordial germ cells (PGCs). In vertebrates, PGCs actively migrate during embryonic development from the position where they are specified toward the site of the genital ridge, where they will differentiate into gametes with the unique role of transmitting genetic information between generations [1,2].

Studies on mouse and zebrafish germ cells show that during migration, chemokine receptor-4 (CXCR4), a seven-transmembrane G protein-coupled receptor, and its ligand, stromal cell-derived factor-1 (SDF1), are involved in the proliferation, motility, and survival of PGCs, gonocytes, and spermatogonial stem cells [3,4]. CXCR4 plays roles in a variety of biological processes, including development, the immune response, and tumorigenesis [5,6,7]. There is a growing amount of genetic evidence showing that CXCR4 controls many types of normal and pathological cell migrations. CXCR4 and its ligand are responsible for the maintenance of adult stem cell niches, stimulate the invasive migration of testicular germ cell tumor (TGCT) cell lines *in vitro*, and selectively influence seminoma migration and metastasis [8,9,10]. In zebrafish embryos, PGCs are formed before gastrulation, specified in four different locations, repulsed by somatic cells in regions vacated by germ cells, attracted by the signals that originate at their target locations, and migrate toward the positions where the gonads develop [1]. During this process, PGCs express the chemokine receptor CXCR4b, an ortholog of mammalian CXCR4, and are attracted toward the gonads, where the CXCR4b ligand SDF1a is highly expressed. An increase in signaling by the overexpression of CXCR4b, or a decrease by its knockdown, disrupts normal PGC migration, causing PGCs to arrive at ectopic positions [11], and disrupting the normal distribution of CXCR4b on the plasma membrane can trigger similar PGC homing phenotypes [12].

Reticulons (RTNs) are a group of evolutionarily-conserved proteins present in plants, yeast, and vertebrates [13,14,15]. Almost all members of this group contain a highly-conserved carboxy-terminal reticulon homology domain (RHD) that consists of two hydrophobic membrane structures, and their amino-terminal domains are variable and rarely similar [16,17,18]. In mammals, the reticulon protein family comprises four proteins (RTN1, RTN2, RTN3, and RTN4/Nogo) [18,19,20], that are widely expressed in most tissues, especially in human and mouse brains [20,21]. Previous studies on RTN3 focused on how RTNs modulate neuronal regeneration [21], axonal development [22], monocyte recruitment, atherosclerosis [23], and neurodegenerative diseases such as Alzheimer’s disease [24]. Although the functions of RTN3 in apoptotic signal transduction and cell death have been studied intensively [25,26], there are no reports on the contribution of RTN3 to PGC migration.

In this study, we identified RTN3 as an important component guiding PGC migration in zebrafish. We showed that RTN3 could directly interact with CXCR4, regulate the expression level of CXCR4 and alter the localization of CXCR4 at the plasma membrane in HEK293 and HeLa cells, suggests a mechanism how RTN3 affects PGC migration in zebrafish.

## 2. Results

### 2.1. CXCR4 Can Interact with the C-Terminal RTN Homolog Domain of RTN3

**Figure 1:**
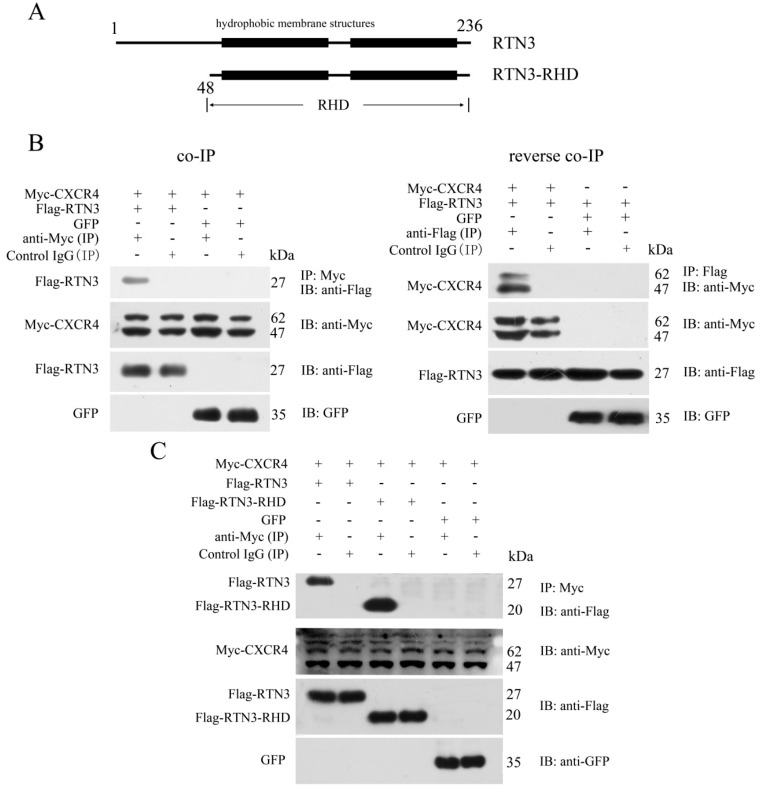
Specific interaction of CXCR4 with the RHD of RTN3. (**A**) Schematic representation of RTN3 and RTN3-RHD. Amino acid residues 48 to 236 comprise the RHD of RTN3; (**B**) The interaction between Myc-CXCR4 and Flag-RTN3. Myc-hCXCR4, Flag-hRTN3, and GFP were overexpressed in HEK293 cells. Cell lysates were immunoprecipitated with anti-Myc or anti-Flag or control IgG antibodies, separated by SDS-PAGE and immunoblotted with the corresponding antibodies; (**C**) CXCR4 interacted with the RHD of RTN3. Myc-hCXCR4, Flag-hRTN3, Flag-hRTN3-RHD, and GFP were overexpressed in HEK293 cells. Cell lysates were immunoprecipitated with anti-Myc or control IgG antibodies followed by immunoblotting with the corresponding antibodies.

To identify proteins that interact with CXCR4 and potentially affect its functions in PGC migration, we used the human full-length CXCR4 protein as bait to screen a human fetal liver cDNA library using the yeast two-hybrid system. After bait self-activation testing, an RTN3 fragment containing amino acid residues 86 to 236 was separated from the cDNA library as an interacting partner (Figure 1A). To verify the interaction between CXCR4 and RTN3 detected by the yeast two-hybrid screen, a co-immunoprecipitation (co-IP) assay was performed. The results showed that CXCR4 directly interacted with RTN3, and the reverse co-IP assay confirmed this interaction. In the co-IP assay, pcDNA3.1-Myc**-**hCXCR4 was transiently co-transfected into HEK293 cells with pCMV-Flag**-**hRTN3 or pEGFP-N1. The results of this assay showed that Flag-RTN3, but not the GFP control, formed a stable complex with Myc-CXCR4 and allowed it to be precipitated with an antibody recognizing Myc-CXCR4 but not with a control IgG. Furthermore, the reverse co-IP assay verified that Flag-RTN3 interacted with Myc-CXCR4, but not the GFP control and was recognized by the anti-Flag antibody but not the control IgG (Figure 1B).

RTN3 contains a variable N-terminus and a highly-conserved C-terminal RTN homology domain. Most of the RTN3 fragments isolated by the yeast two-hybrid screen were included in the RHD. We, therefore, investigated whether the RHD of RTN3 played an important role in the interaction with CXCR4. We constructed RTN3-RHD as an RTN3 deletion mutant (Figure 1A). Myc-tagged CXCR4 and either Flag-tagged full-length RTN3 or the Flag-tagged RTN3-RHD mutant were overexpressed in HEK293 cells. Subsequent co-IP and immunoblotting revealed the interaction between the RHD of RTN3 and CXCR4 (Figure 1C). Taken together, CXCR4 can interact with the RHD of RTN3.

### 2.2. RTN3 Regulates the CXCR4 Expression Level in HEK293 Cells

**Figure 2:**
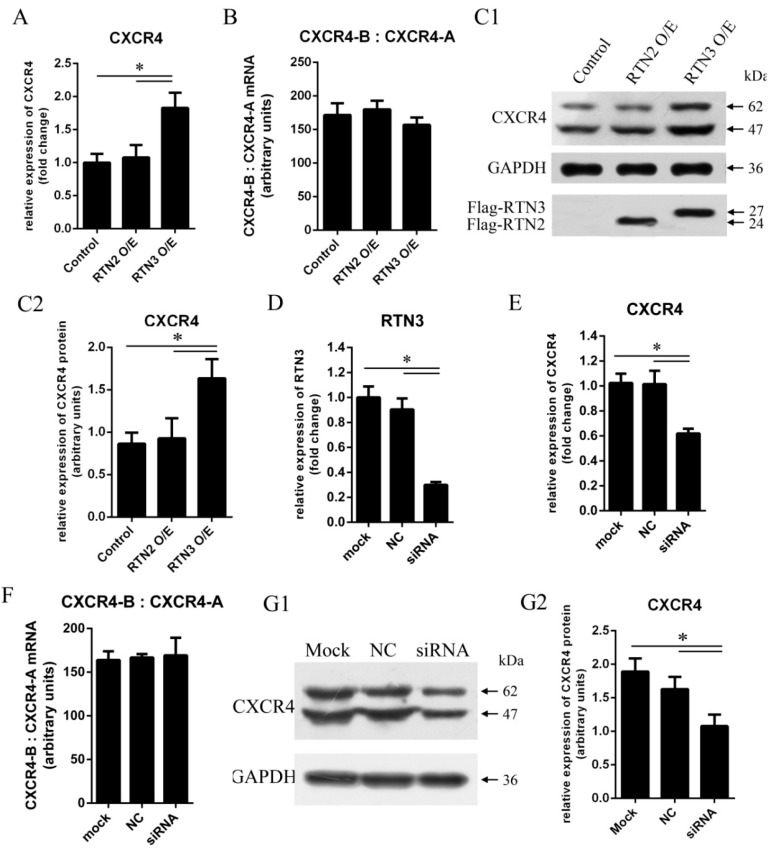
CXCR4 expression levels are elevated by RTN3 overexpression and suppressed by RTN3 deficiency. (**A**, **C1**, **C2**, **E**, **G1**, **G2**) Endogenous CXCR4 mRNA and protein expression in HEK293 cells. Endogenous CXCR4 or GAPDH mRNA and protein levels were monitored by real-time PCR and Wwestern blotting; **C2** and **G2** show quantitative analyses of the grayscale band intensity of the western blots; (**B**, **F**) Relative expression of the CXCR4-B and CXCR4-A mRNA isoforms. The ratio between CXCR4-B and CXCR4-A mRNAs was detected by real-time PCR in HEK293 cells when the RTN3 expression level was changed; (**D**) RTN3 knockdown in HEK293 cells. Small interfering RNAs (siRNAs) targeting RTN3 were transfected into HEK293 cells for 48 h, and endogenous RTN3 mRNA as detected by real-time PCR to determine the efficiency of RTN3 knockdown. Data represent the mean ± SEM from three wells per group. * *p* < 0.05. Results are representative of three independent experiments.

To determine whether RTN3 could regulate CXCR4 expression, HEK293 cells were transfected with pCMV-Flag-hRTN3 encoding human RTN3 protein fused to a Flag tag or with an empty vector as a control. To verify that the effect on CXCR4 is specific to RTN3, we used RTN2 as another control. CXCR4 mRNA expression was detected using quantitative real-time PCR at 48 h post-transfection, and the endogenous expression of CXCR4 protein was detected by western blotting at 72 h post-transfection. Transfection of cells with RTN3 caused the mRNA and protein expression levels of CXCR4 to be modestly higher than in cells transfected with empty vector or RTN2 (Figure 2A,C1,C2), demonstrating that upregulation of RTN3 can elevate the mRNA and protein expression levels of CXCR4 in HEK293 cells.

In addition, we investigated whether downregulation of RTN3 could inhibit the mRNA and protein expression of CXCR4. RNA interference (RNAi) was used to inhibit endogenous RTN3 expression. Small interfering RNAs (siRNAs) for RTN3 were transfected into HEK293 cells to inhibit endogenous RTN3 expression, and the non-silencing control siRNA was not complementary to RTN3 (Figure 2D). Specific inhibition of RTN3 resulted in significant decreases in the mRNA and protein expression levels of CXCR4 (Figure 2E,G1,G2). We also investigated the ratio of the physiological expression of the two isoforms of human CXCR4 mRNA, CXCR4-A, and CXCR4-B, as measured by quantitative real-time PCR in the cells in the experimental and control groups. The proportions of CXCR4-A and CXCR4-B were not significantly changed (Figure 2B,F). Consequently, these results demonstrate that RTN3 induced changes in CXCR4 gene expression in HEK293 cells.

### 2.3. Subcellular Localization of CXCR4 is Altered by RTN3 in HeLa Cells

**Figure 3:**
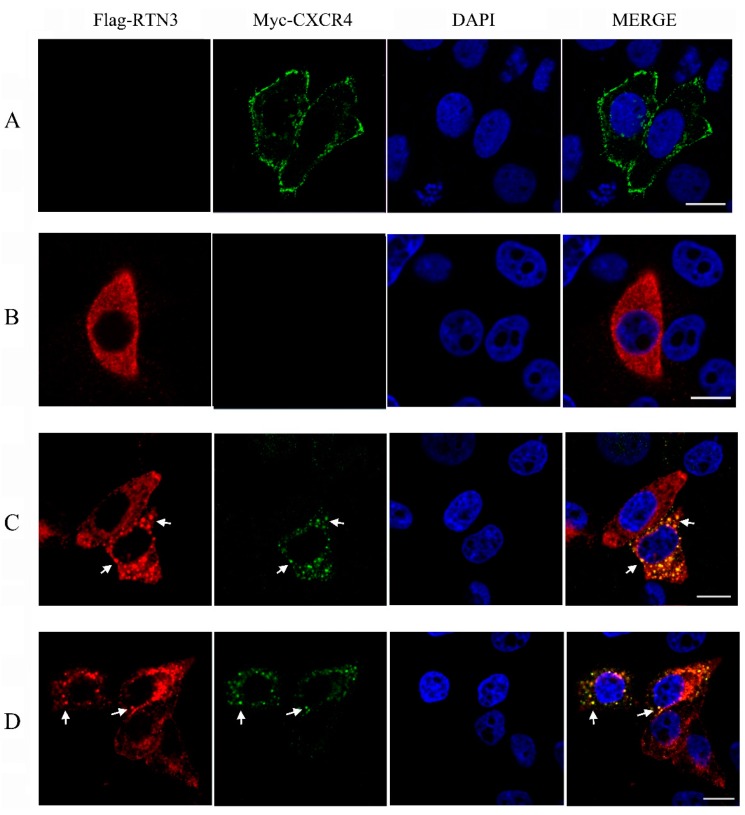
Translocation of CXCR4 in HeLa cells. (**A**,**B**) The subcellular localization of hCXCR4 and hRTN3. HeLa cells were transfected with pcDNA3.1-Myc-hCXCR4 (**A**) and pCMV-Flag-hRTN3 (**B**); (**C**,**D**) Translocation and colocalization of CXCR4 and RTN3. HeLa cells were co-transfected with human Myc-CXCR4 and Flag-RTN3 (**C**) or with zebrafish Myc-CXCR4b and Flag-RTN3 (**D**). After 48 h, the transfected cells were fixed and subjected to immunofluorescence assay using anti-Myc mouse monoclonal antibody (mAb), anti-Flag mAb, and 4′,6-diamidino-2-phenylindole (DAPI). The results of subcellular localization were screened using confocal laser-scanning microscopy. Arrows indicate puncta in HeLa cells. Scale bar, 10 µm.

To investigate the subcellular localization of CXCR4 and RTN3, an immunofluorescence assay was performed in HeLa cells. The eukaryotic expression plasmids pcDNA3.1-Myc-hCXCR4 encoding Myc-tagged human CXCR4 and pCMV-Flag-hRTN3 encoding Flag-tagged human RTN3 were overexpressed in HeLa cells, and the localization patterns were screened using confocal microscopy (Zeiss, Dresden, Germany). When HeLa cells were separately transfected with CXCR4 or RTN3, CXCR4 protein expression was mainly localized on the plasma membrane in HeLa cells, and the predominant subcellular localization of RTN3 was diffusely distributed in the cytoplasm (Figure 3A,B). However, after co-transfection, the subcellular localization of CXCR4 was altered, and RTN3 translocation was observed, with the two proteins typically co-localized as punctate structures in the cytoplasm in co-transfected cells (Figure 3C). These results indicate that RTN3 directly interacts with CXCR4 and mediates its sub-cellular localization.

To investigate whether overexpressing the zebrafish CXCR4 and RTN3 produces a similar effect, pcDNA3.1-Myc-zCXCR4 and pCMV-Flag-zRTN3, encoding the zebrafish CXCR4b and RTN3 genes, which are homologous to the human genes, were co-transfected into HeLa cells. Similar levels of colocalization and translocation of CXCR4 and RTN3 were observed (Figure 3D), indicating that zCXCR4b and zRTN3 may show an interaction similar to that of hCXCR4 and hRTN3, with similar effects on the biological function of CXCR4.

### 2.4. Enhanced Signaling of RTN3 Results in PGC (Primordial Germ Cell) Migration Defects Similar to Those Caused by CXCR4b Overexpression in Zebrafish Embryos

**Figure 4:**
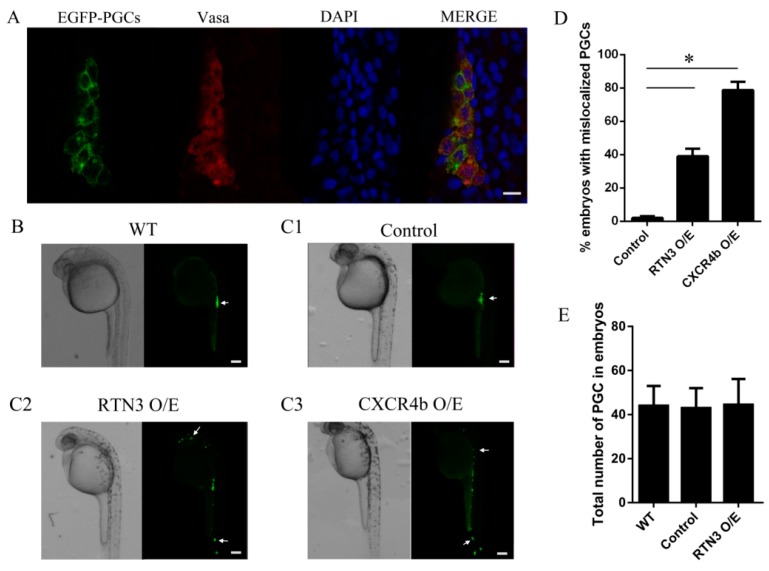
The level of RTN3 expression plays a role in PGC migration to the gonadal region in zebrafish. (**A**) Specificity analysis of the PGCs labeled with EGFP. Embryos were collected at 30 h post-fertilization (hpf), and whole-mount fluorescence immunohistochemistry was performed using an anti-Vasa antibody to specifically label the germ cells; (**B**) PGC migration in wild-type embryos. Wild-type embryos were collected at about 24 hpf, oriented in agarose on a confocal dish, and screened using confocal microscopy; (**C**) Ectopic PGCs in RTN3 and CXCR4b mRNA injected embryos. Embryos were injected at the one-cell stage with 300 pg CXCR4b or 250 pg RTN3 synthetic mRNA per embryo. For controls, 1 nL of rhodamine was injected. After approximately 30 h, embryos were collected, and the positions of the PGCs were observed using confocal microscopy; (**D**) The percentages of embryos with mislocalized PGCs. Increasing the level of RTN3 or CXCR4b expression affected the number of ectopic PGCs compared with the control group; (**E**) Quantitative analysis of the numbers of PGCs in the experimental and control embryos. A total of 50–100 embryos were analyzed for each group. Error bars represent the mean ± SEM, * *p* < 0.05. Results are representative of three independent experiments. Scale bar, 20 μm for panel **A** and 100 μm for panels **B**, **C1**, **C2**, and **C3**. The arrows in **B** and **C** indicate PGCs.

CXCR4b activation is essential for PGC directional migration, and based on the data shown above, we investigated whether RTN3 could impact the biological function of CXCR4 and lead to mislocalized PGCs. For this purpose, we used zebrafish from the kop-EGFP-nos1-3′UTR transgenic fish line, with specific expression of EGFP in the PGCs, to investigate the function of RTN3 in PGC migration. To confirm the specificity of the EGFP labeling of PGCs, fluorescence immunohistochemistry was performed with an antibody against zebrafish Vasa protein, which is a specific gene marker of germ cells. These results showed that the EGFP-labeled cells were PGCs (Figure 4A). Next, we increased CXCR4b or RTN3 translation by injecting CXCR4b or RTN3 mRNA, respectively, such that the mRNA concentrations did not obviously affect the general embryonic development into 1-cell embryos, and examined the effect of this overexpression on PGC migration in zebrafish.

To ensure that all PGCs reached their destination, we observed PGC distribution at approximately 30 h post-fertilization (hpf) instead of 24 hpf, when PGCs had already migrated to the gonad in wild-type embryos (Figure 4B). These results showed that, unlike the control embryos, in which PGCs reached the gonad region, embryos injected with RTN3 mRNA had PGCs randomly distributed throughout the body, in ectopic positions. A similar phenotype was observed in the embryos injected with CXCR4b mRNA, in which PGCs lost the correct migratory pathway and were seldom found at the proper position (Figure 4C1–C3). Increasing the level of RTN3 increased the frequency at which PGCs were observed in ectopic locations (Figure 4D), but the numbers of PGCs were not significantly changed (Figure 4E). Together, our results suggest that the level of RTN3 signaling plays a role in PGC migration to the gonad, as seen with CXCR4b in zebrafish.

## 3. Discussion

In this report, we investigated the interaction between RTN3 and CXCR4 as well as the potential function of RTN3 in PGC migration in zebrafish. We demonstrated that CXCR4 directly interacts with the RHD domain of RTN3 and that RTN3 mediates endogenous CXCR4 expression, changes the subcellular localization of CXCR4, and co-localizes with CXCR4. We also observed that increased RTN3 signaling could disturb PGC migration and could increase localization at ectopic positions. This phenotype of PGC migration defects is similar to the effect of aberrantly expressed CXCR4b in zebrafish. Taken together, our data suggest that RTN3 can modulate CXCR4 signaling and mediates PGC movement to the region of the developed gonad.

Members of the RTN family were previously considered as candidate modulators that play important roles in apoptotic signal transduction and cell death. The RTN family may represent the only molecules to participate in all three apoptosis signaling pathways, the extrinsic pathway, intrinsic pathway and endoplasmic reticulum (ER)-stress pathway, and RTN proteins have demonstrated a strong tendency for aggregation. RTN3 can form homodimers or heterodimers with RTN4 and can promote or suppress apoptosis [27]. In addition, RTN3 can also interact with Bcl-2, CRELD1, and FADD, which do not belong to the RTN family [26,28,29]. In our study, we observed that RTN3 interacts with CXCR4, a seven-transmembrane G protein-coupled receptor, using yeast two-hybrid and co-IP assays, indicating that RTN3 may perform certain biological functions via binding with CXCR4.

Almost all RTN family members contain variable amino termini that do not display significant sequence similarity and a highly conserved carboxy-terminus named the RHD. The divergent amino terminus plays specific roles in mammals, showing species and tissue specificity, whereas the RHD may execute more basic cellular functions [30]. Among mammals, the RHDs of RTNs 1, 3, and 4 have an average 73% sequence identity at the amino acid level, and RTN2 has approximately 52% sequence similarity [16]. Therefore, we hypothesized that RTN3 could interact with CXCR4 through its highly conserved carboxy-terminal domain. The results from co-IP are consistent with this hypothesis: CXCR4 interacted with the RTN3 carboxy-terminus, suggesting that the RHD of RTN3 plays a role in its interaction with CXCR4.

CXCR4 plays important roles in immune trafficking and in the migration of cells including neurons, germ cells, cancer cells, and other cell types. Dysregulated expression of CXCR4 directly results in disruptions of stem cell homing and neuronal migration [31]. We investigated whether RTN3 participates in the process of PGC migration via CXCR4 induction. Our data showed that the CXCR4 mRNA and protein expression levels were significantly increased by RTN3 overexpression, which indicates that RTN3 dysregulation might impair CXCR4 function.

Moreover, multiple forms of CXCR4 with different molecular weight (MW) exist in mammalian cells; there are numerous post-translational modifications that can generate this MW heterogeneity, including *N*-glycosylation, disulfide formation, oligomerization, and proteolysis. However, different MW forms of CXCR4 might perform different biological functions. For instance, Sloane *et al.* [32] found 11 different MW forms of CXCR4 in CEMT4 cells, but only the 83-kDa form has a biological function in human immunodeficiency virus (HIV) infection. Duquenne *et al.* [33] showed that there are two isoforms of CXCR4 in humans, CXCR4-A and CXCR4-B. CXCR4-A encodes a longer N-terminus than the CXCR4-B isoform by including the first nine amino acid residues. However, although only CXCR4-B in HOS cells plays a role in HIV-1 infection, the responses of the two isoforms to SDF1, their natural ligand, were indistinguishable. In our studies, we found that changing the level of RTN3 signaling level did not significantly change the ratio of the two isoforms of CXCR4 in HEK293 cells, suggesting that both CXCR4 isoforms responded to RTN3 (Figure 2B,F).

Previous studies have suggested that the level of CXCR4 signaling is important for proper migration of PGCs and that CXCR4 subcellular localization is also necessary for PGC homing [12]. We examined the subcellular localization of CXCR4 in RTN3-transfected HeLa cells. After transient co-transfection with CXCR4 and RTN3, CXCR4 expression on the plasma membrane was sharply reduced, and multiple cytoplasmic vesicles were formed. These vesicles strongly co-localized with RTN3. As in zebrafish, CXCR4b underwent translocalization in response to RTN3 co-transfection in HeLa cells. These results demonstrate that the subcellular localization of zebrafish and human CXCR4 resulted from RTN3-mediated receptor activation.

To confirm the functional significance of RTN3 in guided PGC migration in zebrafish, we evaluated the capacity of PGCs guided by RTN3 to arrive at the gonad. The PGCs of the embryos overexpressing RTN3 were found at random sites throughout the embryos and formed distinctly-dispersed cell clusters rather than the compact clusters found at the gonad in wild-type embryos. Similar results were obtained when CXCR4b was overexpressed in embryos.

Our study indicates that RTN3 interacts with CXCR4 directly and regulates CXCR4 expression and localization in HEK293 cells. Furthermore, the expression of particularly high levels of RTN3 results in imprecise cell migration in zebrafish, which indicates that RTN3 participates in the process of PGC homing based on CXCR4b-induced migration in zebrafish.

The primary role of RTN3 maybe to contribute to PGC migration. Considering that we did not observe any obvious change in PGC number, the overexpression of RTN3 may not affect PGC survival. One possible mechanism by which RTN3 may regulate PGC migration is by changing the subcellular localization of CXCR4 to affect cell polarity and motility. This hypothesis requires confirmation through further experiments, such as measuring the distance and speed of PGC migration during zebrafish embryonic development using a time-lapse camera [31,34]. The effects of RTN3 on PGC migration may be cell autonomous or non-autonomous. Our current data are insufficient to explain the mechanisms of PGC migration. If RTN3 is expressed in PGCs and affects the distribution of CXCR4 in PGCs, and the migration process does not depend on surrounding somatic cells, then the effect may autonomous, but RTN3 may also be expressed in the somatic cells around the PGCs, meaning that the migration process may be dependent on the surrounding somatic cell, then it could be non-autonomous. The mechanisms by which RTN3 affects PGC migration require further confirmation through cell transplantation experiments [4,35,36]. These possible mechanism are intended more to indicate a possible direction for our future work.

## 4. Experimental Section

### 4.1. Plasmid Construction

The open reading frames (ORFs) of hCXCR4 and hRTN3 were separately cloned from HEK293 cell cDNA using the primer pairs hCXCR4-F, hCXCR4-R, hRTN3-F, hRTN3-R, hRTN2-F, and hRTN2-R based on the human CXCR4 (GenBank accession no. BC020968.2), RTN3 (NM_006054.3), and RTN2 (NM_206901.2) sequences. The zCXCR4b and zRTN3 ORFs were cloned from cDNA isolated from zebrafish embryo cDNA using the primer pairs zCXCR4b-F, zCXCR4b-R, zRTN3-F, and zRTN3-R based on the zebrafish CXCR4b (AF201451.1) and RTN3 (NM_201072.1) sequences. Detailed information about the primer pairs is provided in Table 1.

pGBKT7-hCXCR4 was constructed by cloning hCXCR4 into the expression vector pGBKT7 (Clontech, California, CA, USA), which encoded the full-length CXCR4 fused to the GAL4 DNA-binding domain for yeast two-hybrid screening. pcDNA3.1-Myc-hCXCR4 and pcDNA3.1-Myc-zCXCR4b were obtained by the respective cloning of human and zebrafish CXCR4b genes into the mammalian expression vector pcDNA3.1-Myc (Invitrogen, California, CA, USA) to express human CXCR4 or zebrafish CXCR4b fused to a N-terminal Myc epitope tag. pCMV-Flag-hRTN3, pCMV-Flag-hRTN2, and pCMV-Flag-zRTN3 were created by the respective cloning of human RTN3, RTN2, and zebrafish RTN3 genes into the mammalian expression vector pCMV-N-Flag (Beyotime, Shanghai, China) encoding human RTN3, RTN2, or zebrafish RTN3 fused to a N-terminal Flag epitope tag. pCMV-Flag-hRTN3-RHD was constructed using standard PCR-subcloning techniques; subcloned PCR products were amplified from pCMV-Flag-hRTN3 using the primer pairs hRTN3-RHD-F and hRTN3-R and were subsequently inserted into the pCMV-N-Flag vector. pEGFP-N1 empty vector (Clontech) encoding enhanced green fluorescent protein (EGFP), and was used as a control. pGEM-zCXCR4 and pGEM-zRTN3 were constructed by cloning zCXCR4 and zRTN3 into the transcription vector pGEM-4Z (Promega, Madison, AL, USA).

### 4.2. siRNA Design

For knockdown of human RTN3 in HEK293 cells, three small interfering RNAs (siRNAs) were designed by Suzhou GenePharma Co., LTD (Suzhou, China). The specific siRNAs sequence is 5′-CCACUCAGUCCCAUUCCAUTT-3′, 5′-GGAUCUACAAGUCCGUCAUTT-3′, and 5′-CCUUCUAAUUCUUGCUGAATT-3′. Non-silencing control siRNA was designed with a 21-nt random sequence, 5′-UUCUCCGAACGUGUCACGUTT-3′, which does not match within the human genome.

### 4.3. Cell Culture and Transfection

HEK293 and HeLa cells were cultured in Dulbecco’s modified Eagle’s medium (DMEM) basic supplemented with 10% FBS and antibiotics in 5% CO_2_ at 37 °C. The cells were plated in 12-well, six-well, or 10 cm plates, transfected with the expression vectors using Lipofectamine 2000 (Invitrogen) reagent and maintained in the complete medium.

### 4.4. Yeast Two-Hybrid Screening

The AH109 yeast strain transfected with pGBKT7-hCXCR4 was used to screen a human fetal liver cDNA library cloned into the GAL4 activation domain vector (pACT2, Clontech, California, CA, USA). Details of the methods have been described previously [37].

### 4.5. Co-Immunoprecipitation and Immunoblotting

HEK293 cells were lysed in radioimmunoprecipitation assay (RIPA) buffer containing 25 mM Tris base-HCl (pH 7.5), 150 mM NaCl, 1.0 mM EDTA (pH 8.0), 1.0 mM EGTA (pH 8.0), 1% NP-40, 0.5% sodium deoxycholate, protease inhibitor cocktail (Roche, Basel, Switzerland), and phosphatase inhibitor cocktail (Roche). After removing the insoluble debris by centrifugation at 12,000 rpm for 15 min at 4 °C, the supernatants were subjected to immunoprecipitation with anti-Flag M2 Affinity Gel (Sigma, Shanghai, China) or Anti-Myc Mouse Monoclonal Antibody Agarose Resin (CWBIO, Beijing, China) overnight at 4 °C. After five washes with RIPA buffer, the bound protein was eluted with either sodium dodecyl sulfate (SDS) sample buffer or elution buffer (0.2 M glycine pH 2.5, neutralized with 1.5 M Tris pH 9.0). Proteins were separated by SDS-polyacrylamide gel electrophoresis (SDS-PAGE) and were transferred onto nitrocellulose (NC) membranes (Millipore, Darmstadt, Germany). The immunoblot analyses were conducted with relative antibodies: mouse monoclonal against Flag (Sigma), Myc (Abcam, Cambridge, England), GFP (CWBIO), or GAPDH (CWBIO), goat polyclonal against CXCR4 (Santa Cruz, Dallas, TX, USA), and horseradish peroxidase (HRP)-labeled secondary antibodies.

### 4.6. Quantitative Real-Time PCR

Total RNA was extracted using TRIzol (Invitrogen) reagent. Reverse transcription was performed using the GoScript reverse transcription system (Promega). The cDNA was amplified with specific primers in a LightCycler 480 (Roche) with SYBR Green. The specific primers hRTN3-RT-F and hRTN3-RT-R for human RTN3 were designed using Primer Express 3.0 (Table 1), and the specific primers for human GAPDH, CXCR4, CXCR4-A, and CXCR4-B were described previously [33,38,39]. Real-time quantitative PCR was performed under the following cycling conditions, 95 °C for 10 min for pre-denaturation, followed by 40 cycles of 95 °C for 15 s and 60 °C for 1 min, then 95 °C for 15 s, 60 °C for 15 s, and 95 °C for 15 s to acquire a melting curve for the PCR product to confirm the amplification specificity. All reactions were conducted in triplicate. The relative mRNA abundances were calculated utilizing the 2^−ΔΔ*C*t^ method with GAPDH or β-actin as the reference and plotted as the fold change compared with the control or mock samples.

### 4.7. mRNA Synthesis and Microinjection

Capped sense RNAs were synthesized from linearized pGEM-zCXCR4 and pGEM-zRTN3 *in vitro* using a mMessage Machine Kit (Ambion, California, CA, USA). Zebrafish embryos were injected at the one-cell stage with 1 nL per injection. Synthetic mRNA was microinjected at a nominal concentration of 300 pg per embryo (CXCR4b) or 250 pg per embryo (RTN3). For controls, 1 nL of Rhodamine B (Sigma) was injected per embryo.

### 4.8. Zebrafish and Transgenic Line

Zebrafish (*Danio rerio*) of the AB genetic background were maintained and raised as previously described [3,4]. The kop-EGFP-nos1-3′UTR transgenic fish line in which PGCs could be visualized *in vivo* was a gift from Chen at the Chinese Academy of Sciences. The animal welfare and experimental procedures adhered to the Institutional Guidelines of the Care and Use of Laboratory Animals at China Agricultural University (Beijing, China). All efforts were made to minimize suffering.

### 4.9. Immunofluorescence and Microscopy

HeLa cells were washed with phosphate-buffered saline (PBS), fixed with 4% paraformaldehyde for 10 min, permeabilized with 0.5% (*v*/*v*) Triton X-100 in PBS for 10 min, and blocked with 0.5% bovine serum albumin (BSA) in PBS. Zebrafish embryos were collected and fixed with 4% paraformaldehyde for 4 h at room temperature, permeabilized with 1% (*v*/*v*) Triton X-100 in PBS for 30 min, and blocked with PBTA (1.5% BSA and 0.3% Tween-20 in PBS) for 1 h. The cells or embryos were then successively stained with anti-Flag or anti-Myc antibodies, fluorescein isothiocyanate (FITC) or Cy3-conjugated secondary antibodies (CWBIO), and 4′,6-diamidino-2-phenylindole (DAPI, Sigma). Then, images were captured using a Zeiss microscope (LSM 510, Zeiss, Dresden, Germany). The embryos were oriented in 1.5% low-melting-pointagarose on a confocal dish (coverglass bottom dish) and were visualized on a Zeiss microscope.

### 4.10. Statistical Analysis

Statistical analyses were performed using SPSS 16.0 (IBM, Armonk, NY, USA). Data are presented as the means ± SEM and were obtained from at least three independent experiments. The mean relative changes in the mRNA levels were compared among treatment groups and control or mock groups using the paired Student’s *t*-test. Values of *p* ≤ 0.05 were considered significant.

## 5. Conclusions

By screening CXCR4-interacting proteins using a yeast two-hybrid approach, we have identified a new CXCR4-interacting proteins, RTN3, which can regulate CXCR4 expression and translocation. Moreover, increased RTN3 signaling in zebrafish can disrupt normal PGC migration. These results indicate that the level of RTN3 expression may play a role in PGC migration through interaction with CXCR4.

## Figures and Tables

**Table 1:** Primers used in this study.

Name	Sequence (5′ to 3′)
hCXCR4-F	ATGGAGGGGATCAGTATATA
hCXCR4-R	TTAGCTGGAGTGAAAACTTG
hRTN3-F	ATGGCGGAGCCGTCGGCGGC
hRTN3-R	TTATTCTGCCTTTTTTTTGG
hRTN2-F	ATGGGGAGTAAAGTGGCGGA
hRTN2-R	TCATTCGGCTTTGGCTTTGG
zCXCR4-F	ATGGAATTTTACGATAGCA
zCXCR4-R	ACTCGTCAGTGCACTGGAC
zRTN3-F	ATGGCAGACCCAATGACCC
zRTN3-R	TCATTCTGCTTTACAACGCT
hRTN3-RT-F	CCATCCATTCAAAGCCTACCTG
hRTN3-RT-R	CACCAACATAGGTCATCAGCC
hRTN3-RHD-F	GTGCACGATCTGATTTTCTG
